# Intracranial Arteriovenous Malformation Combined with Multiple Aneurysms Diagnosed by CTA: A Case Report

**DOI:** 10.5334/jbsr.3006

**Published:** 2023-03-06

**Authors:** Qin Zhang, Yongzhe Hou, Qiong Wang, Xilong Chen

**Affiliations:** 1 Gansu University of Traditional Chinese Medicine, CN; 2 The Third People’s Hospital of Bijie City, Radiology Department, CN; 3 Department of PICU, Gansu Provincial Maternity and Child-Care Hospital, CN

**Keywords:** arteriovenous malformation, aneurysm, computed tomography angiography, intracranial, cerebral

## Abstract

Arteriovenous malformation (AVM) combined with aneurysms is not uncommon, but AVM of the basilar artery, brainstem, and right middle cerebral artery combined with multiple intracranial aneurysms (IAs) is rare. Cases of aneurysm protrusion into the optic canal are also rare. We report a distinctive case of intracranial AVM combined with multiple IAs and partial protrusion of a cavernous segment aneurysm of the right internal carotid artery into the optic nerve canal.

**Teaching Point:**

Cases of partial protrusion of a cavernous segment aneurysm of the right internal carotid artery into the optic canal, resulting in widening of the optic canal compared to the contralateral side, compression, thickening and swelling of the subocular veins, and obstruction of venous drainage warrant the clinician’s attention.

## Introduction

Intracranial arteriovenous malformation (AVM) combined with multiple intracranial aneurysms (IAs) is a complicated and specific cerebrovascular disease, with an incidence ranging from 2.7% to 16.7% and is a common risk factor for hemorrhage, with a high mortality rate [1].

## Case History

**Figure 1:**
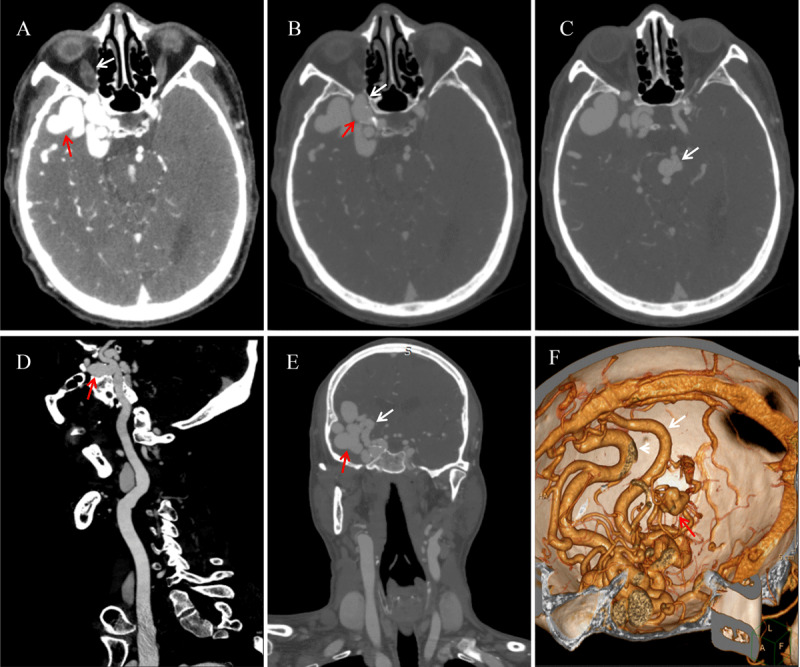
CTA presentation of intracranial arteriovenous malformation combined with multiple aneurysms.

A 57-year-old man was rushed to the emergency room with intermittent convulsions. The patient had a five-year history of hypertension and a single seizure one year earlier. Axial computed tomography angiography (CTA) image (Figure 1A) showed a vascular mass (red arrow) and thickened, swollen inferior ophthalmic vein (white arrow). The bone window axial CTA image (Figure 1B) demonstrated a right internal cavernous sinus aneurysm locally protruding into the optic canal (red arrow) and optic canal enlargement compared to the contralateral side (white arrow). A basilar artery aneurysm was present (white arrow in Figure 1C). Multiplanar reformation sagittal image (Figure 1D) shows saccular aneurysm (red arrow) approximately 2.4 cm × 1.3 cm in size with a narrow neck originating from the cavernous segment of the right internal carotid artery. The bone window coronal CTA image (Figure 1E) demonstrates the feeding artery (white arrow) and the malformed vascular mass (red arrow), while the volume rendering (Figure 1F) illustrates the draining vein (white arrow) and the basilar artery aneurysm (red arrow).

The final diagnosis was intracranial AVM combined with multiple IAs and a right internal carotid artery aneurysm. The patient was given antiepileptic, vasospasm-relieving, and blood pressure-controlling medication after being admitted to the hospital. When the patient had stabilized, the patient was transferred to another hospital. However, due to financial constraints, the patient and his family declined the further therapy and were self-discharged from the hospital.

## Comments

The mechanism of AVM combined with IA is complicated. IA mostly occurs in the supplying arteries of AVM and there is a blood flow correlation between IA and AVM. Moreover, fibrous degeneration and thinning of the AVM-supplying arterial walls, disruption of the intima, and alterations in the vascular musculature lead to a significant increase in pressure in the AVM-supplying vessel. These hemodynamic mechanisms may explain the development of AVM combined with IA.

Given the patient’s imaging data, this case of AVM combined with IA is difficult to handle because of the following characteristics:

- IA occurred in the supplying artery of the AVM, which is different from the vascular structure of an ordinary IA and is clinically rare.
- Besides the IA in the intracranial arteriovenous malformation mass, IA was also present in the distal basilar artery and the right internal carotid artery.
- The IA of the cavernous segment of the right internal carotid artery partially protruded into the optic canal, resulting in widening of the optic canal compared to the contralateral side and compression, thickening, and swelling of the inferior ophthalmic vein by obstruction of venous outflow.
- The arteriovenous malformation vascular mass was supplied by the C5–6 segment of the internal carotid artery and the basilar artery, and several drainage veins were seen converging into the superior sagittal sinus. And
- IA predominantly occurred in vessels other than ordinary IA vessels (e.g., anterior and posterior communicating arteries).

Digital subtraction angiography (DSA), which is the gold standard for the clinical diagnosis of AVM and IA, its use is restricted because it is an invasive examination and more likely to cause cerebrovascular accidents than CTA (e.g., aneurysm rupture and transient cerebral ischemia due to vasospasm). However, because CTA is a noninvasive, inexpensive, and quick imaging technique, it can be utilized as a reliable alternative to DSA in emergencies demanding immediate operation [2]. CTA and its multiple reconstruction modalities (e.g., volume rendering, maximal-intensity projection, multiplane reconstruction) are of great value in the diagnosis of AVM and IA. The clinical significance of CTA is that it can reveal the blood supply arteries and drainage veins of AVM, as well as the location, size, morphology, thrombi, and calcification of AVM, allowing physicians to more precisely assess AVM and IA. CTA may also display the architecture of the lesion and its relationship with neighboring tissues in numerous angles and planes. Additionally, the CTA technique can save examination time, accurately localize bleeding, and help with disease characterization when acute rupture of IA occurs, guiding the development of treatment plans. Furthermore, patients prefer to select less expensive CTA in some poor areas of developing countries. Therefore, diagnoses of AVM combined with IA can be made using CTA as an alternate imaging technique to DSA.

## Conclusion

We reported a rare case of intracranial AVM combined with multiple IAs and partial protrusion of a cavernous segment aneurysm of the right internal carotid artery into the optic nerve canal, to increase awareness of this rare cerebrovascular disease.
